# Using interactive Jupyter Notebooks and BioConda for FAIR and reproducible biomolecular simulation workflows

**DOI:** 10.1371/journal.pcbi.1012173

**Published:** 2024-06-20

**Authors:** Genís Bayarri, Pau Andrio, Josep Lluís Gelpí, Adam Hospital, Modesto Orozco

**Affiliations:** 1 Institute for Research in Biomedicine (IRB Barcelona), the Barcelona Institute of Science and Technology, Barcelona, Spain; 2 Barcelona Supercomputing Center (BSC), Barcelona, Spain; 3 Department of Biochemistry and Biomedicine, University of Barcelona, Barcelona, Spain; bioinformatics.ca, CANADA

## Abstract

Interactive Jupyter Notebooks in combination with Conda environments can be used to generate FAIR (Findable, Accessible, Interoperable and Reusable/Reproducible) biomolecular simulation workflows. The interactive programming code accompanied by documentation and the possibility to inspect intermediate results with versatile graphical charts and data visualization is very helpful, especially in iterative processes, where parameters might be adjusted to a particular system of interest. This work presents a collection of FAIR notebooks covering various areas of the biomolecular simulation field, such as molecular dynamics (MD), protein–ligand docking, molecular checking/modeling, molecular interactions, and free energy perturbations. Workflows can be launched with myBinder or easily installed in a local system. The collection of notebooks aims to provide a compilation of demonstration workflows, and it is continuously updated and expanded with examples using new methodologies and tools.

## Introduction

Scientific research is inexorably moving to an open and reproducible format [1]. Increased research transparency and reproducibility have a direct effect on the credibility of the scientific literature published. Reproducibility in computational science has greatly improved in the last few years [2,3], mainly boosted by the data science community [4]. Life sciences and computational biology research have recently started to adopt these methodologies [5–8]. Furthermore, a new movement intending to adapt the Findable, Accessible, Interoperable and Reusable/Reproducible (FAIR) data principles [9] to research software development has emerged [10–13]. Using standards and state-of-the-art tools, this software FAIRification ensures not only reproducibility, but also *Findability* (how to find), *Accessibility* (how to access), *Interoperability* (how to integrate), and *Reusability* (how to use).

Regrettably, the field of biomolecular simulation is known for its lack of standards and best practices, thus hindering the generation of open, interoperable, and reproducible science. Several recent initiatives have attempted to start community-driven processes to define best practices for software and workflow development and data sharing in the field [6,14–16]. The BioExcel Centre of Excellence for Computational Biomolecular Research (BioExcel CoE, https://bioexcel.eu/) is leading one such initiative. BioExcel Building Blocks (BioBB) [17] is a new computational library with particular attention to interoperability, accessibility, and reproducibility/reusability in biomolecular simulation workflows. Designed following the recommendations of the ELIXIR European Life Science infrastructure and the FAIR principles for software development, the library provides a set of interoperable units based on a collection of Python wrappers encapsulating software components. It is freely accessible from GitHub and BioConda packages [18], installable via Conda, Docker and Singularity containers, and compatible with several workflow languages [19].

Jupyter Notebooks are documents shared in an open-source web application (computational notebook), and they are compatible with live code, equations, visualizations, and text. They are now routinely used for documentation purposes in code development processes [20], openness/reproducibility [21,22], and for hands-on teaching [23–26]. During the COVID-19 pandemic (2020), they emerged as fantastic tools for online training events, with the possibility to use them as tutorials comprising interactive programming code accompanied by text information and/or documentation, versatile graphical charts, and data visualization. Furthermore, the combination of Jupyter Notebooks with easy installation of software dependencies using packaging systems such as Conda [27] makes these workflows highly shareable and reproducible.

Here, we present a collection of reproducible, FAIR, easy-to-install, and highly documented Jupyter Notebooks implementing common biomolecular simulations workflow tutorials, developed with the combination of the BioBB library and BioConda packages, ready to be used for training purposes.

## Results/Evaluation

**Fig 1:**
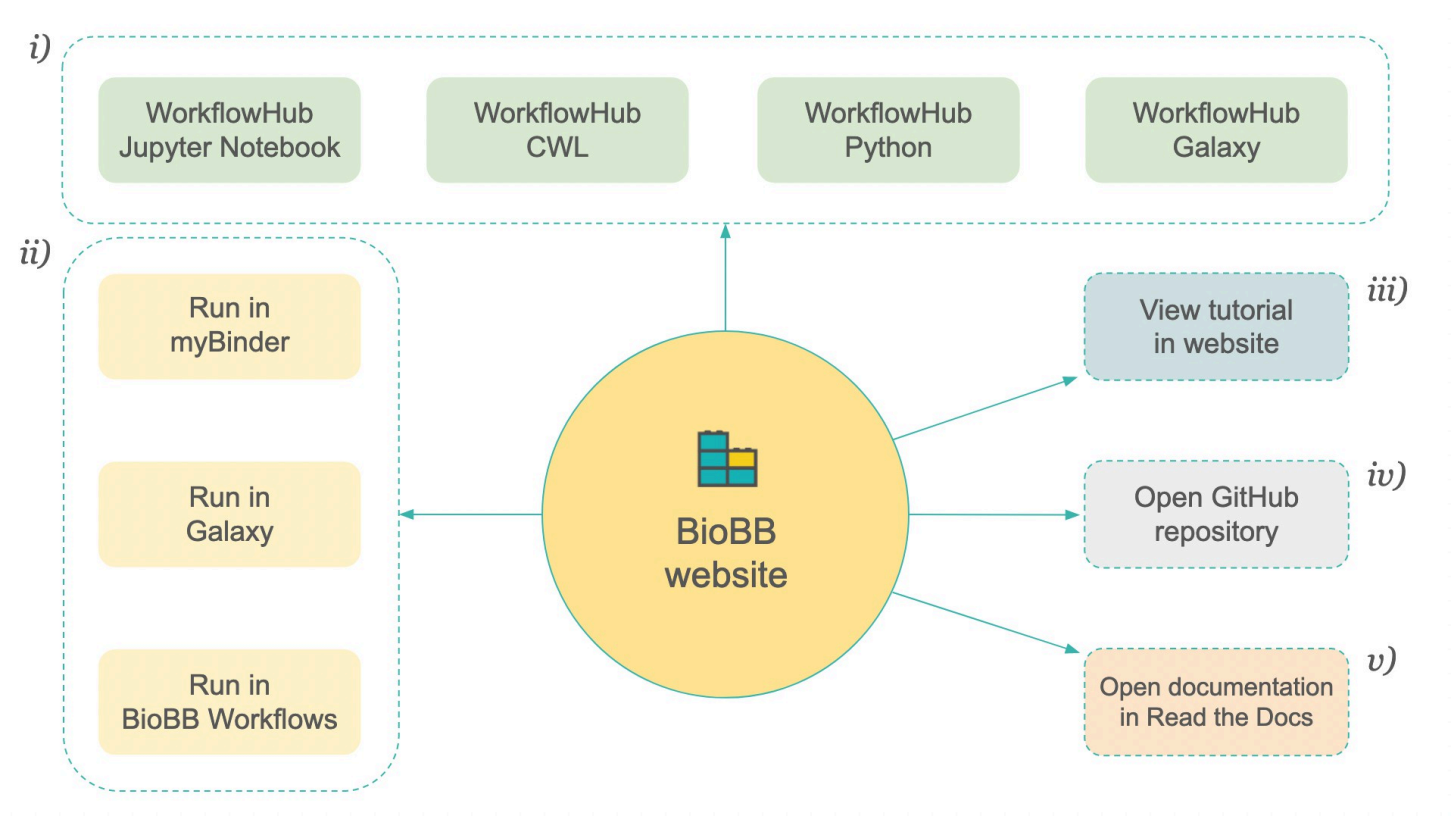
Information available on the BioBB website for the workflow tutorials, including links to: (i) workflow versions registered and available in WorkflowHub (Jupyter Notebook, CWL, Python and Galaxy); (ii) ways to directly launch the workflow (Jupyter Notebook myBinder, Galaxy and BioBB Workflows [28] website); (iii) tutorial in web version (HTML); (iv) source code in GitHub; and (v) documentation in Read the Docs.

The collection of Jupyter Notebooks can be accessed from the workflows section of the main BioBB website: https://mmb.irbbarcelona.org/biobb/workflows. Each tutorial has an independent block with a short description, version, WorkflowHub entries, available deployment sites, source code, and documentation (Fig 1).

The current version (2024.1) provides a collection of 17 workflows covering various areas of the biomolecular simulation field, including molecular dynamics (MD), protein–ligand docking, molecular checking/modeling, molecular interactions, and free energy perturbations. An exhaustive list of available workflows and where they can be found is given in Table A in S1 Data.

**Fig 2:**
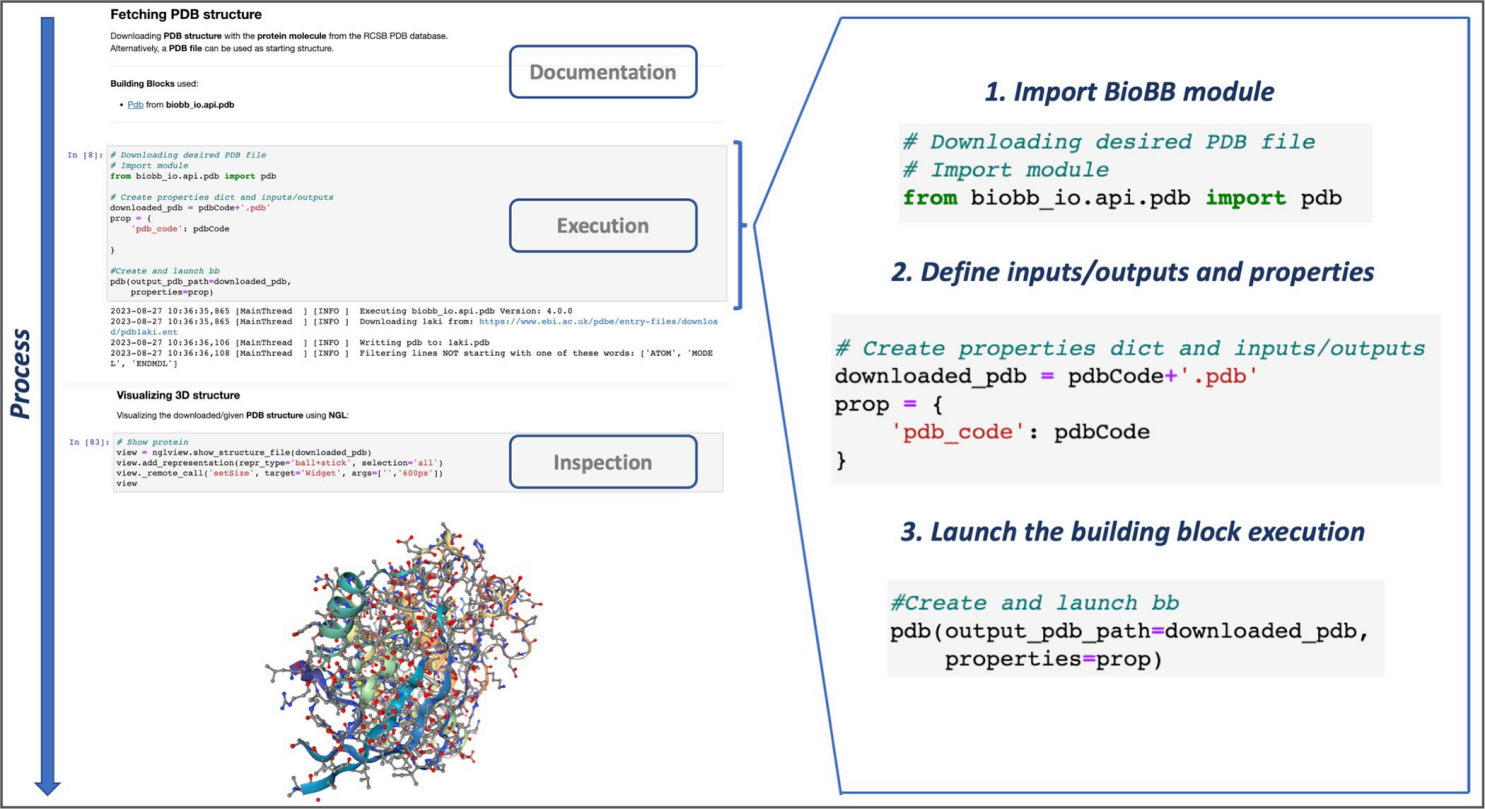
Uniformity in the Jupyter Notebooks collection: in the pipeline process, with markdown cell for documentation followed by the execution cell and the graphical inspection of the intermediate results (left); and in the cell execution, using the BioBB syntax of importing the module, defining inputs/outputs and properties, and launching the execution for all processes run in the workflow (inline, right).

All the notebooks share a uniform header, with a title and description, a list of the BioBB modules and auxiliary libraries used, the command lines required to install and launch the workflow, and a clickable index of the pipeline steps (Fig A in S1 Data). Following this header, the pipelines encoded in the notebooks are fragmented and explained step by step. Each step process is enclosed in a Jupyter cell, preceded by documentation about what the step is doing, and followed by a graphical inspection of intermediate results whenever possible (Fig 2). This format allows the practice of reading, understanding, and executing the pipeline gradually, moving from top to bottom.

In addition, the use of the BioBB library makes the workflows easier to understand, as all execution step cells follow the same uniform syntax (Fig 2 inline). Each of these cells imports a specific building block, defines the inputs/outputs (as regular disk files) and properties (method variables) of the tool wrapped, and finally launches the execution.

To demonstrate the utility of our interactive Jupyter Notebooks, 4 distinct examples, briefly described in the following sections, were selected.

### Example 1: Protein MD setup (GROMACS)

**Target audience**: Users interested in MD simulations with little or even no knowledge about the method.

**Examples of use**: Prepare (setup) and run your first MD simulation for a small globular protein structure using the GROMACS MD package.

**GitHub URL**: https://github.com/bioexcel/biobb_wf_md_setup

The first example tutorial [29] aims to illustrate the process of setting up an MD simulation system containing a protein with GROMACS MD engine [30], using the Lysozyme protein (PDB code 1AKI) as input. The workflow is strongly based on the pipeline included in the collection of MD tutorials written by Justin Lemkul and available on-line here: https://www.mdtutorials.com/gmx/lysozyme/index.html [31].

**Fig 3:**
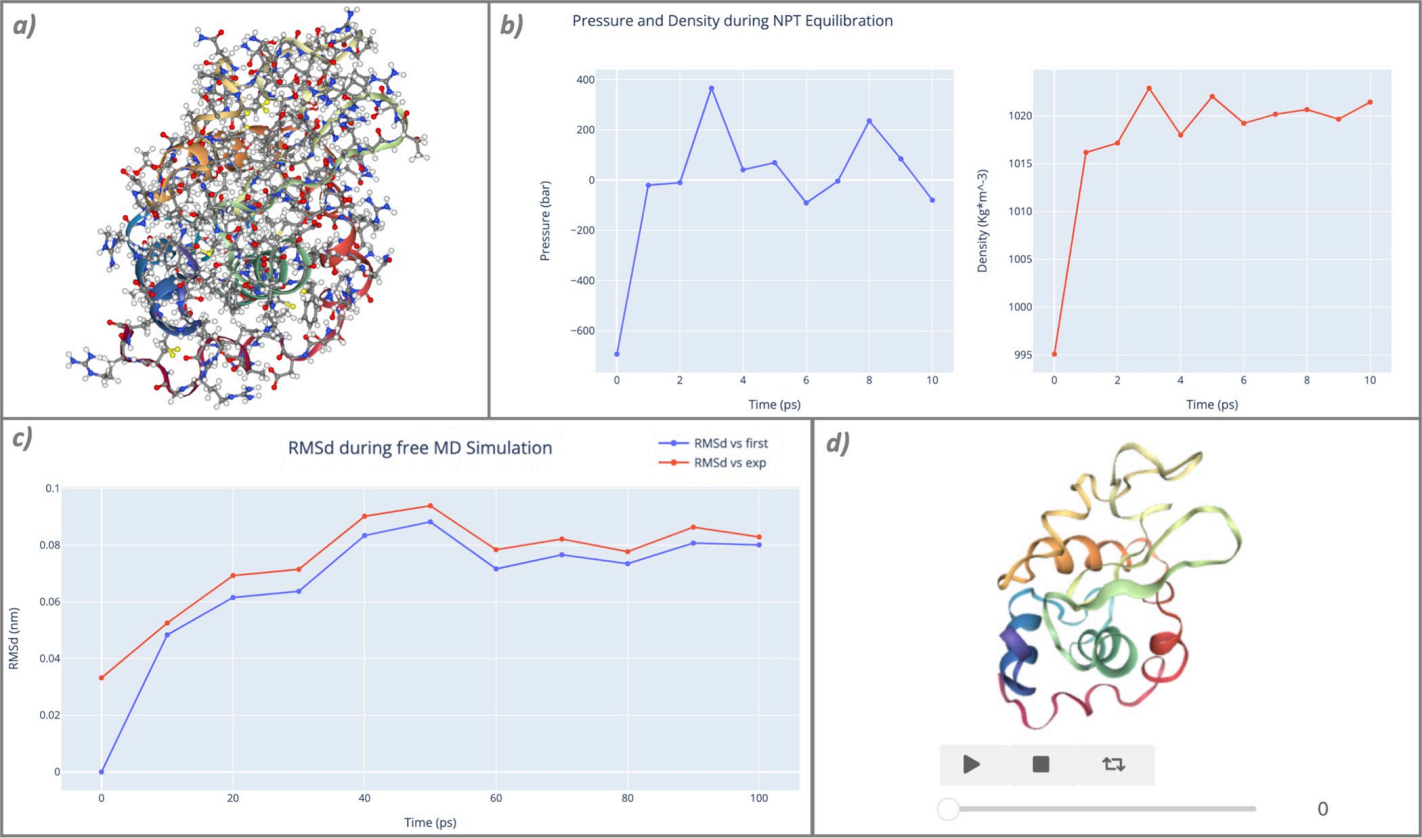
Intermediate results extracted from the “GROMACS Protein MD Setup” workflow. (a) PDB structure with missing atoms added, ready to be used as MD input; (b) pressure and density box parameters measured over time in the final NPT equilibration process (10ps); (c) RMSd of the snapshots included in the final trajectory against the first snapshot (blue) and the original experimental structure (red); and (d) interactive visualization of the final trajectory using the simpletraj tool.

The workflow is divided into 14 sections, including retrieving the PDB structure from the PDB database [32], preparing the structure (Fig 3A), developing an MD topology and system box with explicit water molecules and counterions, energetically minimizing and equilibrating the system (Fig 3B), running a short, unrestrained MD simulation, and finally post-processing (Fig 3C) and visualizing the short trajectory generated (Fig 3D).

Inspection of intermediate results (Fig 3) is a useful functionality in Jupyter Notebooks. As an example in this particular case, visualization of the outputs for the energy minimization or system equilibration process is a direct indicator of convergence problems in the system energy and/or box, which could suggest that these processes should be extended to allow the system to relax. The final root mean square deviations (RMSd) can also denote that the simulation needs more time to converge.

Associated with the convergence, it is noteworthy that the time scales used in this demonstration workflow are shorter than those typically used in the field, for the sake of time in running the whole workflow. For example, a common equilibration process can easily reach tens of nanoseconds, whereas a common production MD can go up to the millisecond time scale. Although these variables can be easily adjusted in the workflow, it is important to understand that these notebooks are designed to be used as demonstrators and not as production scripts.

### Example 2: Protein–ligand docking

**Target audience**: Users interested in protein–ligand docking processes with little or even no knowledge about the method.

**Examples of use**: Find possible binding sites on the surface of a protein structure, explore the suitability for a particular ligand to dock at one of the discovered regions, and obtain a final predicted protein–ligand complex structure.

**GitHub URL (Protein–ligand docking workflows)**:

https://github.com/bioexcel/biobb_wf_virtual-screening

**GitHub URL (Protein–ligand docking workflow, *fpocket* version)**:

https://github.com/bioexcel/biobb_wf_virtual-screening/tree/master/biobb_wf_virtual-screening/notebooks/fpocket

The second example [33] illustrates the process of protein–ligand docking, a prediction of the position and orientation of a small molecule when bound to a protein receptor or enzyme. The particular example used is the Mitogen-activated protein kinase 14 (p38-α) protein (PDB code 3HEC), a well-known protein kinase enzyme, in complex with the FDA-approved Imatinib (PDB Ligand code STI, DrugBank [34] Ligand Code DB00619), a small molecule kinase inhibitor used to treat certain types of cancer. The tutorial guides the user through the process of identifying the active site cavity (pocket) without previous knowledge using fpocket [35], and the final prediction of the protein–ligand complex using the AutoDock Vina program [36].

**Fig 4:**
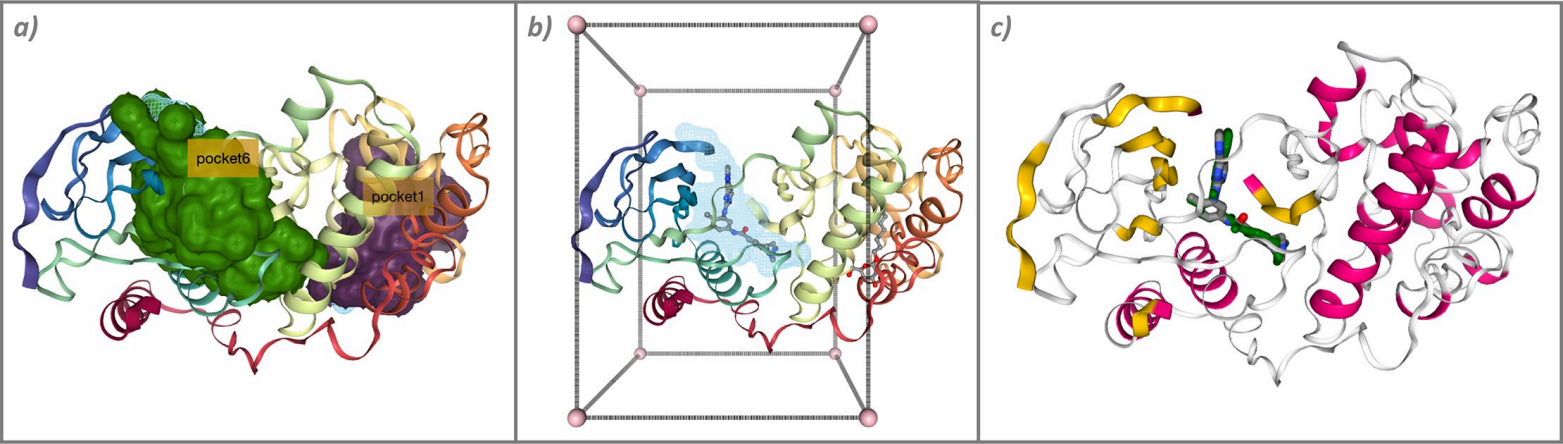
Intermediate results extracted from the “Protein–ligand Docking” workflow. (a) Pockets identified on the surface of the protein by the fpocket tool; (b) box including the protein pocket to be used in the docking process; and (c) final comparison of the chosen ligand pose against the experimental structure.

The workflow is divided into 17 sections, including retrieving the PDB structure from the PDB database, computing the protein cavities (Fig 4A), generating a box around a selected cavity (Fig 4B), downloading the ligand, preparing the 2 molecules (protein and ligand) for the docking process (adding atomistic charges and formatting the files), running the docking task, extracting a chosen docking pose, superposing the final ligand pose to the target protein structure, and comparing the final result with an existing experimental structure to validate the workflow (Fig 4C).

This workflow adds more interactivity than the previous one, including the possibility for the user to choose the pocket to include in the final docking run, and also the ligand pose to keep as a final result. These selections are implemented as dropdown lists in the Jupyter Notebook. However, this interactivity makes the workflow unsuitable to be used in an unsupervised way, as stated at the beginning of the notebook with a warning banner.

### Example 3: Protein coarse-grained flexibility

**Target audience**: Users interested in fast (coarse-grained) theoretical methods to explore protein dynamics and flexibility with little or even no knowledge about the methods.

**Examples of use**: Generate a set of predicted 3D conformations from a determined protein and explore the main flexible regions and global dynamics without spending too much time and computational resources.

**GitHub URL**: https://github.com/bioexcel/biobb_wf_flexserv

The third tutorial [37] aims to illustrate the process of generating protein conformational ensembles from 3D structures and analyzing their molecular flexibility. The notebook reproduces the workflow integrated into the FlexServ web-based tool for the analysis of protein flexibility [38]. The workflow incorporates powerful protocols for the coarse-grained (CG) determination of protein dynamics using different versions of Normal Mode Analysis (NMA), Brownian dynamics (BD), and Discrete Dynamics (DMD). It also integrates a set of flexibility analyses using a large variety of metrics, including basic geometrical analysis, B-Factors, essential dynamics, stiffness analysis, collectivity measures, Lindemann’s indexes, residue correlation, chain-correlations, dynamic domain determination, and hinge point detections, all of them computed with the pcasuite tool [39,40]. The particular structure used is the Ribosomal Protein S15 from Bacillus Stearothermophilus (PDB code 1A32).

**Fig 5:**
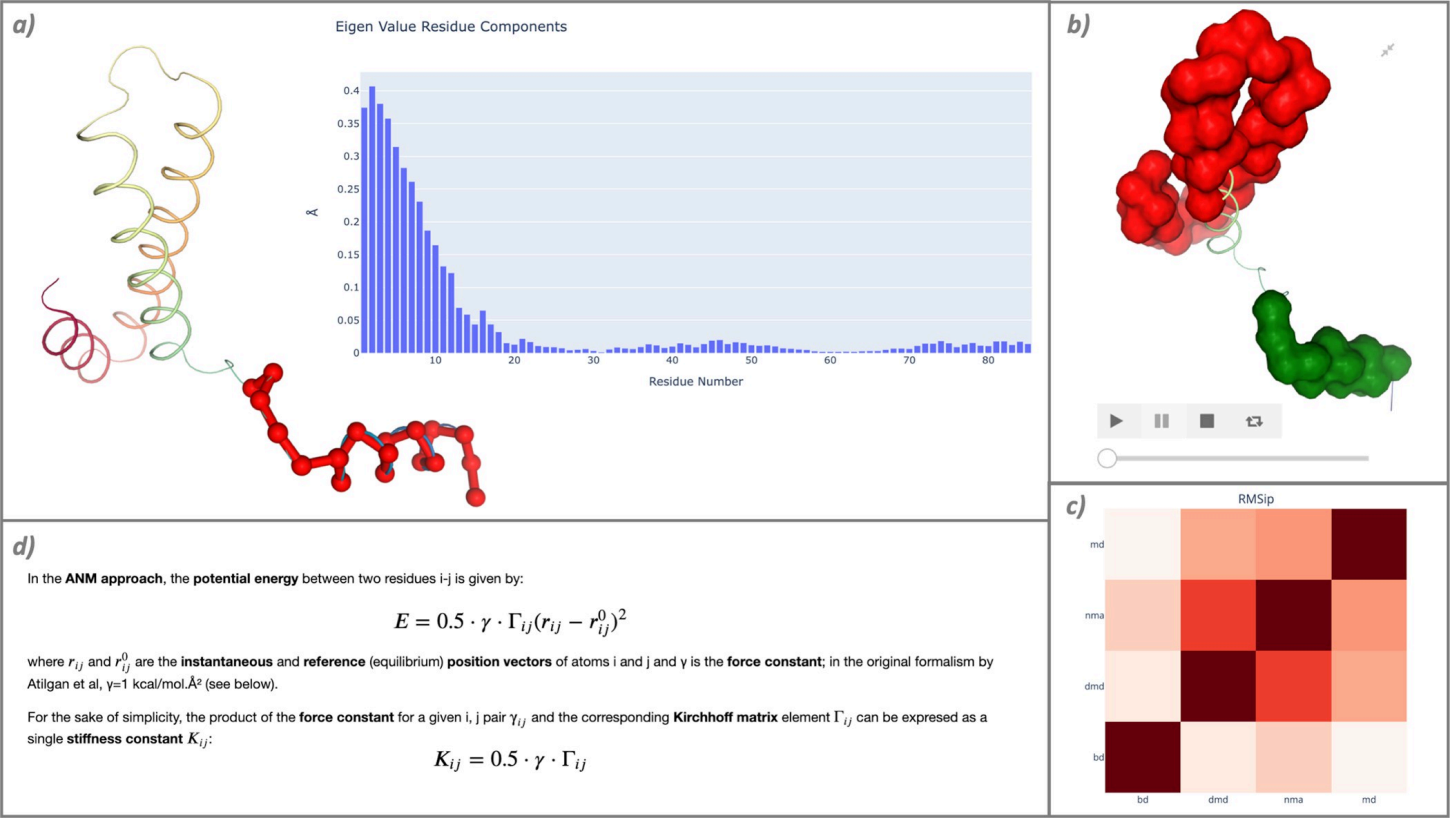
Markdown documentation and intermediate results extracted from the “Macromolecular Coarse-Grained Flexibility” workflow. (a) Eigenvalue residue components analysis, indicating the residues contributing the most to the key essential deformations of the protein. An associated NGL widget displays these residues in ball and stick representation (red); (b) domain decomposition analysis displayed along the pseudo-trajectory with the simpletraj tool; (c) RMS inner product (RMSip) for all the CG pseudo-trajectories against an atomistic MD simulation; and (d) extract of documentation included for the NMA CG method.

The workflow is divided into 2 main sections, namely CG generation of conformational ensembles, and flexibility analysis of the resulting pseudo-trajectories. The first part involves computing the ensembles using 3 CG methods: NMA, BD, and DMD while the second part uses the PCA statistical method to extract the essential dynamics [41] from the ensembles and obtain a collection of flexibility descriptors, which are graphically represented using NGL [42,43] and plotly [44] tools. The features obtained from these analyses facilitate the identification of the protein flexible domains through the components of the eigenvalue (Fig 5A), B-Factors, or hinge point prediction (Fig 5B). To graphically examine how far the pseudo-trajectories fall from covering the protein conformational landscape explored by MD, the workflow includes a final comparison of all the CG-generated ensembles with an atomistic MD simulation, (Fig 5C).

One particularity of this workflow is the rich markdown documentation included. Each CG method is complemented by an extended explanation, with equations embedded in mathematical notation (Fig 5D). Therefore, the notebook offers a reference to the CG methods basics, while simultaneously giving the user the possibility to run and explore the generated ensembles.

### Example 4: Molecular interaction potentials

**Target audience**: Users interested in exploring molecular interactions (protein–ligand, protein–protein) for a particular system. Basic knowledge about electrostatic interactions is recommended.

**Examples of use**: Place a first shell of water molecules in the energetically most favorable positions of the protein surface; explore the interaction properties of a protein surface regions through the affinity for negative, positive, or hydrophobic residues; identify the residue/atom which is contributing the most to a particular protein–ligand or protein–protein complex interaction, and evaluate the interaction energy.

**GitHub URL**: https://github.com/bioexcel/biobb_wf_cmip

The last tutorial used to exemplify the collection of workflows aims to illustrate the process of computing classical molecular interaction potentials from protein structures [45]. It includes molecular interaction potential (MIP) grids, protein–protein/ligand interaction potentials, and protein titration. The particular structures used are the Lysozyme protein (PDB code 1AKI), the Epidermal Growth Factor Receptor kinase domain (PDB code 4HJO) complexed with the Erlotinib inhibitor (PDB code AQ4), and an MD simulation of the complex formed by the SARS-CoV-2 Receptor Binding Domain and the human Angiotensin Converting Enzyme 2 (PDB code 6VW1). The software tool wrapped by the BioBB library and used in this workflow is called Classical Molecular Interaction Potentials (CMIP) [46].

**Fig 6:**
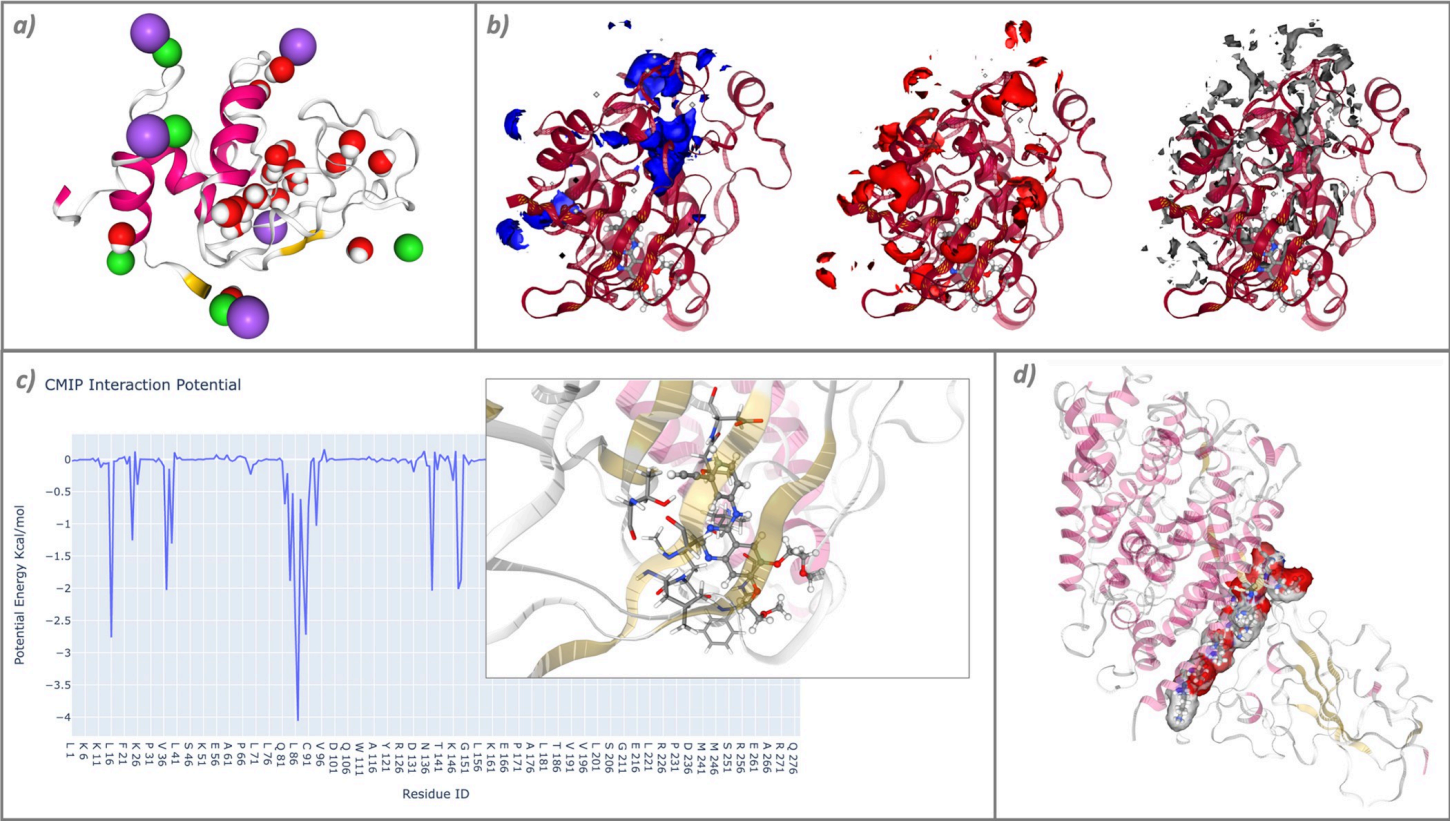
Intermediate results extracted from the “Molecular Interaction Potentials” workflow. (a) Structural water molecules and ions placed in the energetically most favorable spots on the surface of the protein; (b) molecular interaction potential grids obtained from a positive probe (left, blue), a negative probe (middle, red), and a neutral probe (right, gray); and (c) potential energy (electrostatic + VdW) calculated for a protein–ligand interaction. The inline plot shows the representation of the protein residues with lower energy (higher affinity). (d) Representation of the residues contributing the most to the protein–protein complex interaction.

The workflow is divided into 3 main sections: structural water molecules and ions (titration), MIPs, and interaction potential energies. In the first part, the pipeline shows how to place a desired number of water molecules and ions in the energetically most favorable spots on the surface of the protein, a useful method for the preparation of input systems for MD simulations (Fig 6A). In the second part, molecular interaction potential grids are generated with 3 distinct electro-charged probes: positive, negative, and neutral. The representation of the resulting grids allows graphical exploration of regions of the proteins with affinity for negative, positive, or hydrophobic residues (respectively) (Fig 6B). Finally, in the third part, examples of how to compute molecular interaction energies in protein–ligand and protein–protein complexes are presented. The method uses the Poisson–Boltzmann equation to calculate the electrostatic potential by default, although all parameters can be tuned. The results are parsed and energies accumulated by residue, thereby allowing for easy identification of the residues with the greatest contribution to the interaction (Fig 6C). The interaction region can also be easily identified and represented with a simple energy cutoff (Fig 6D).

## Discussion/Conclusions

The FAIRification of computational biomolecular simulation workflows following the principles presented by initiatives like FAIR4RS [10] is starting to promote reproducibility and reusability in the field. We believe that the collection of workflows presented herein is a good example. They fulfill the most important rules for FAIR workflow implementation [47]: workflows are properly registered and described with rich metadata (Findability); source code is available in public code repositories and example input data and results are provided along with the workflows (Accessibility); tools integrated into the workflows are interoperable between them thanks to the BioBB library (see Methods) and are adhering to file format standards (Interoperability); and finally they come with an associated reproducible computational environment to run the workflow, are flexible with parameter tuning and compatible with input configuration files, are highly modular (BioBB building blocks), with the possibility to easily swap building blocks and reconfigure the workflow, contain clear and concise documentation included as markdown cells, and include qualified references to the software used through a list of citations (Reusability/Reproducibility). All these points are explained in detail in the Materials and methods section.

Two of these FAIR points are especially relevant for our field: the possibility to extensively document the workflow and the high degree of reusability/reproducibility offered by the packaging systems used. The combination of these led to a collection of workflows prepared to be used in training sessions and hands-on tutorials, with students acknowledging: (i) not having to deal with any software dependency installation; (ii) having detailed associated documentation for each of the steps of the workflow; (iii) interactively working with the workflow; (iv) graphically inspecting the intermediate results; and (v) having the possibility to modify/expand an existing workflow.

The possibility to use myBinder to automatically install and execute Jupyter Notebooks in the cloud is highly convenient for the free deployment of these types of demonstration workflows, as it offers the interactive execution of the workflow with just 1 click. The possibility of installing myBinder engine (BinderHub) locally in a private cloud environment opens the door to personalized Binders, providing more control over the number of computational resources and simultaneous users allowed. Additionally, these technologies are starting to reach HPC centers, connecting the power of supercomputers directly from Jupyter Notebooks, a very important step for our kind of calculations. Finally, similar infrastructures are also provided by the most important cloud providers in the world (Amazon, Google, Microsoft’s Azure), offering some interesting advantages such as the possibility to access GPU cards. However, most of these infrastructures are not compatible with the use of Conda environments, which means that additional packages and software dependencies need to be installed inside the workflow and reinstalled at the start of every session, thus hindering reproducibility aspects.

Finally, the presented workflows are transversal, common, and useful pipelines that can be applied as sub-workflows in more complex studies or different platforms. In fact, these demonstration workflows were converted, when possible, to different formats, thanks to the compatibility of the BioBB library with a range of workflow managers [19]. Common Workflow Language (CWL) [48], Galaxy [49], and pure Python versions of the presented workflows are available from a centralized GitHub repository (https://github.com/bioexcel/biobb_workflows/). From these, pure Python scripts, in combination with HPC workflow managers such as PyCOMPSs [50,51], were used in real scientific cases [52,53]. We would like to stress here that, in contrast to these last examples used in HPC environments, the notebooks presented in this work are designed for educational purposes only and not for production usage.

We believe that FAIR and reproducible workflows are the right way to build, execute, and share our scientific pipelines, and we will continue to expand our collection of workflows in the coming years.

## Materials and methods

The digital notebooks presented in this work are strongly reliant on Jupyter Notebooks [54] and Python programming language. The BioBB library [17,19] is the main engine behind the workflows. BioPython library [55] is heavily used in the BioBB modules to process and work with PDB files. NGLview [42], py3Dmol [56], and simpletraj (https://github.com/arose/simpletraj) are used to interactively visualize 3D structures and MD trajectories. Plotly [44] and matplotlib [57] are used to display and plot data with 2D graphs.

Conda (https://docs.conda.io/) is used as a software packaging system. All BioBB modules and associated dependencies (when needed) are packaged with Conda. Conda environments (https://www.anaconda.com/) allow us to make workflows easily reproducible in different machines, using packages from BioConda [18] and conda-forge community channels. Of note, although Conda environments are compatible and can be used with all kinds of Operative Systems (OS), including Apple, Linux, and Windows, the tools used in the workflow (dependencies) are those that will finally determine this compatibility. As an example, if a workflow uses a tool that is compatible only with Linux, the whole workflow will then be compatible only with this specific OS.

MyBinder (https://mybinder.org/) is the technology used to offer the notebooks in an executable and reproducible environment.

GitHub is the chosen repository and version control system to store the digital notebooks. The BioExcel repository (https://github.com/bioexcel) is used to concentrate the collection of BioBB workflows. Each workflow repository contains an associated Conda virtual environment configuration file, including all the needed dependencies that will be automatically installed in the Conda environment. All workflows are also available from the WorkflowHub registry [58], the ELIXIR bioinformatics registry bio.tools [59], and from a central repository with all the available versions of the workflows (Jupyter Notebooks, Common Workflow Language (CWL) [48], pure Python and Galaxy [49]), along with the corresponding installation instructions:

https://github.com/bioexcel/biobb_workflows/.

Read the docs (https://docs.readthedocs.io/) is the technology chosen to document the workflows. All workflows contain an Introduction and installation section and a Tutorial section. All input and output files are linked to their corresponding EDAM ontology [60] (e.g., AMBER parmtop—edam:format_3881). These EDAM terms are also used in the bio.tools entries and CWL specifications.

JavaScript Object Notation for Linking Data (JSON-LD, https://github.com/json-ld/json-ld.org) is used to capture the software references for the BioBB workflows, enclosing a list of citations to all the software that is used in the pipeline. JSON-LD format is a standard-based machine-readable data, in addition to be easy for humans to read and write.

The environmental footprint for a typical run of the presented workflows (1:30 h on 12 CPUs Xeon E5-2683 v4) draws 170.76 Wh. Based in Spain, this has a carbon footprint of 29.21g CO2e, which is equivalent to 0.03 tree-months (calculated using green-algorithms.org v2.2 [61]). Scaling up these workflows to production runs considerably increases the environmental footprint, varying significantly depending on the HPC resources used.

The installation process is shared by all the workflows included in the collection and is based on 4 easy steps:

1. Cloning the GitHub repository (*git clone*).
2. Creating the Conda environment with all required workflow dependencies (*conda env create*). A specific yaml-formatted file is offered for each workflow with the list of required dependencies (tools and libraries).
3. Activating the environment (*conda activate*).
4. Deploying the Jupyter Notebook (*jupyter-notebook*).

Alternatively, a link to myBinder is offered, with a direct deployment, thus avoiding the installation process.

The collection of notebooks is available here:

https://mmb.irbbarcelona.org/biobb/workflows.

Additional links (GitHub, WorkflowHub, bio.tools, and DOIs) are included in S1 Data.

## Supporting information

S1 Data Table A. Collection of FAIR BioBB biomolecular simulation workflows implemented in Jupyter Notebooks. Fig A. Uniform header for all the BioBB tutorials, exemplified with the GROMACS Protein MD setup workflow. Sections included are: (i) title and description; (ii) BioBB modules used; (iii) auxiliary libraries used; (iv) command lines required to install and launch the workflow; and (v) pipeline steps.
(DOCX)
