# Supplementary material for: Using interactive Jupyter Notebooks and BioConda for FAIR and reproducible biomolecular simulation workflows
Source: PLoS Comput Biol. 2024 Jun 20;20(6):e1012173. doi: 10.1371/journal.pcbi.1012173 (PMC11189206; doi:10.1371/journal.pcbi.1012173)
Supplement: S1 Data — Table A. Collection of FAIR BioBB biomolecular simulation workflows implemented in Jupyter Notebooks. Fig A. Uniform header for all the BioBB tutorials, exemplified with the GROMACS Protein MD setup workflow. Sections included are: (i) title and description; (ii) BioBB modules used; (iii) auxiliary libraries used; (iv) command lines required to install and launch the workflow; and (v) pipeline steps. (DOCX) [file pcbi.1012173.s001.docx]

Using interactive Jupyter Notebooks and BioConda for FAIR and reproducible biomolecular simulation workflows

Genís Bayarri^1^, Pau Andrio^3^, Josep Lluís Gelpí^2,3^, Adam Hospital^1*^ and Modesto Orozco^1,2^*

Corresponding authors: [adam.hospital@irbbarcelona](mailto:adam.hospital@irbbarcelona)*, [modesto.orozco@irbbarcelona.org](mailto:modesto.orozco@irbbarcelona.org)*

^1^ Institute for Research in Biomedicine (IRB Barcelona), the Barcelona Institute of Science and Technology, Barcelona, Spain

^2^ Department of Biochemistry and Biomedicine. University of Barcelona. Barcelona, Spain

^3^ Barcelona Supercomputing Center (BSC). Barcelona, Spain

**Supplementary Material**

**Table A**. Collection of FAIR BioBB biomolecular simulation workflows implemented in Jupyter Notebooks

| **Short Title** | **Short Description** | **Programs Used** |
| --- | --- | --- |
| **GROMACS Protein MD Setup** | Illustrates the process of setting up a simulation system containing a protein with GROMACS MD engine | GROMACS[1], Structure_Checking |
| **GitHub:** <https://github.com/bioexcel/biobb_wf_md_setup>  **WorkflowHub**: <https://workflowhub.eu/workflows/120>  **bio.tools**: <https://bio.tools/bioexcel_building_blocks_tutorials_protein_md_setup>  **DOI**: <https://doi.org/10.48546/workflowhub.workflow.120.6> | | |
|  | | |
| **GROMACS Protein-Complex MD Setup** | Illustrates the process of setting up a simulation system containing a protein-ligand complex with GROMACS MD engine | GROMACS[1], Structure_Checking, ACPype[2], OpenBabel[3] |
| **GitHub:** <https://github.com/bioexcel/biobb_wf_protein-complex_md_setup>  **WorkflowHub**: <https://workflowhub.eu/workflows/56>  **bio.tools**: <https://bio.tools/bioexcel_building_blocks_tutorials_protein-ligand_complex_md_setup>  **DOI**: <https://doi.org/10.48546/workflowhub.workflow.56.6> | | |
|  | | |
| **AMBER Protein MD Setup** | Illustrates the process of setting up a simulation system containing a protein with AMBER MD engine | Ambertools[4], Structure_Checking |
| **GitHub:** <https://github.com/bioexcel/biobb_wf_amber_md_setup> (mdsetup notebook)  **WorkflowHub**: <https://workflowhub.eu/workflows/130>  **bio.tools**: <https://bio.tools/bioexcel_building_blocks_tutorials_protein_md_setup_amber_version>  **DOI**: <https://doi.org/10.48546/workflowhub.workflow.130.5> | | |
|  | | |
| **AMBER Protein-Complex MD Setup** | Illustrates the process of setting up a simulation system containing a protein-ligand complex with AMBER MD engine | Ambertools[4], Structure_Checking, ACPype[2], OpenBabel[3] |
| **GitHub:** <https://github.com/bioexcel/biobb_wf_amber_md_setup> (mdsetup_ph notebook)  **WorkflowHub**: <https://workflowhub.eu/workflows/131>  **bio.tools**: <https://bio.tools/bioexcel_building_blocks_tutorials_protein-ligand_complex_md_setup_amber_version>  **DOI**: <https://doi.org/10.48546/workflowhub.workflow.131.5> | | |
|  | | |
| **AMBER Constant pH MD Setup** | Illustrates the process of setting up a simulation system to run constant pH MD simulations with AMBER MD engine | Ambertools[4], Structure_Checking |
| **GitHub:** <https://github.com/bioexcel/biobb_wf_amber_md_setup> (mdsetup notebook)  **WorkflowHub**: <https://workflowhub.eu/workflows/132>  **bio.tools**: <https://bio.tools/bioexcel_building_blocks_tutorials_amber_constant_ph_md_setup>  **DOI**: <https://doi.org/10.48546/workflowhub.workflow.132.5> | | |
|  | | |
| **Automatic Ligand Parameterization** | Illustrates the process of ligand parameterization for a small molecule | ACPype[2], OpenBabel[3] |
| **GitHub:** <https://github.com/bioexcel/biobb_wf_ligand_parameterization>  **WorkflowHub**: <https://workflowhub.eu/workflows/54>  **bio.tools**: <https://bio.tools/bioexcel_building_blocks_tutorials_automatic_ligand_parameterization>  **DOI**: <https://doi.org/10.48546/workflowhub.workflow.54.6> | | |
|  | | |
| **Mutation Free Energy Calculations** | Illustrates the process of computing free energy changes upon protein residue mutations using fast-growth free energy method | GROMACS[1], PMX[5] |
| **GitHub:** <https://github.com/bioexcel/biobb_wf_pmx_tutorial>  **WorkflowHub**: <https://workflowhub.eu/workflows/55>  **bio.tools**: <https://bio.tools/bioexcel_building_blocks_tutorials_mutation_free_energy_calculations>  **DOI**: <https://doi.org/10.48546/workflowhub.workflow.55.6> | | |
|  | | |
| **Protein-Ligand Docking (Cluster90)** | Illustrates the process of protein-ligand docking, using the PDB Cluster90 to obtain the protein pockets | OpenBabel[3],  Structure_Checking, Autodock VINA[6] |
| **GitHub:** <https://github.com/bioexcel/biobb_wf_virtual-screening> (clusterBindingSite notebook)  **WorkflowHub**: <https://workflowhub.eu/workflows/127>  **bio.tools**: <https://bio.tools/bioexcel_building_blocks_tutorials_protein-ligand_docking>  **DOI**: <https://doi.org/10.48546/workflowhub.workflow.127.6> | | |
|  | | |
| **Protein-Ligand Docking (PDBe REST API)** | Illustrates the process of protein-ligand docking, using the PDBe REST-API to obtain the protein pockets | PDBe REST API[7],  OpenBabel[3],  Structure_Checking, Autodock VINA[6] |
| **GitHub:** <https://github.com/bioexcel/biobb_wf_virtual-screening> (ebi_api notebook)  **WorkflowHub**: <https://workflowhub.eu/workflows/128>  **bio.tools**: <https://bio.tools/bioexcel_building_blocks_tutorials_protein-ligand_docking>  **DOI**: <https://doi.org/10.48546/workflowhub.workflow.128.5> | | |
| **Protein-Ligand Docking (fpocket)** | Illustrates the process of protein-ligand docking, using the fpocket tool to obtain the protein pockets | OpenBabel[3],  Structure_Checking, Autodock VINA[6], fpocket[8] |
| **GitHub:** <https://github.com/bioexcel/biobb_wf_virtual-screening> (fpocket notebook)  **WorkflowHub**: <https://workflowhub.eu/workflows/129>  **bio.tools**: <https://bio.tools/bioexcel_building_blocks_tutorials_protein-ligand_docking>  **DOI**: <https://doi.org/10.48546/workflowhub.workflow.129.5> | | |
|  | | |
| **Protein Conformational Ensembles Generation** | Illustrates the process of generating protein conformational ensembles from 3D structures and analyzing its molecular flexibility. A collaboration workflow of BioExcel and ELIXIR 3D-BioInfo structural community. | FlexServ[9], Concoord[10], NOLB[11], iMOD[12], Prody[13], pcasuite[14],  GROMACS[1],  Ambertools[4],  Structure_Checking |
| **GitHub:** <https://github.com/bioexcel/biobb_wf_flexdyn>  **WorkflowHub**: <https://workflowhub.eu/workflows/486>  **bio.tools**: <https://bio.tools/3d-bioinfo_bioexcel_protein_conformational_ensembles_generation>  **DOI**: <https://doi.org/10.48546/workflowhub.workflow.486.3> | | |
|  | | |
| **Macromolecular Coarse-Grained Flexibility** | Illustrates the process of generating protein conformational ensembles from 3D structures using Coarse-Grained tools from the FlexServ server and analysing its molecular flexibility. | FlexServ[9], pcasuite[14],  Structure_Checking,  Ambertools[4] |
| **GitHub:** <https://github.com/bioexcel/biobb_wf_flexserv>  **WorkflowHub**: <https://workflowhub.eu/workflows/551>  **bio.tools**: <https://bio.tools/bioexcel_building_blocks_tutorials_macromolecular_coarse-grained_flexibility>  **DOI**: <https://doi.org/10.48546/workflowhub.workflow.551.2> | | |
|  | | |
| **Molecular Interaction Potentials** | Illustrates the process of computing classical molecular interaction potentials from protein structures | CMIP[15],  Structure_Checking,  Ambertools[4],  ACPype[2] |
| **GitHub:** <https://github.com/bioexcel/biobb_wf_cmip>  **WorkflowHub**: <https://workflowhub.eu/workflows/773>  **bio.tools**: <https://bio.tools/bioexcel_building_blocks_tutorials_molecular_interaction_potentials>  **DOI**: <https://doi.org/10.48546/workflowhub.workflow.773.2> | | |
|  | | |
| **Protein Conformational Transitions Calculations** | Illustrates the process of computing a conformational transition between two known structural conformations of a protein | GOdMD[16],  Structure_Checking,  Ambertools[4] |
| **GitHub:** <https://github.com/bioexcel/biobb_wf_godmd>  **WorkflowHub**: <https://workflowhub.eu/workflows/548>  **bio.tools**: <https://bio.tools/bioexcel_building_blocks_tutorials_protein_conformational_transitions>  **DOI**: <https://doi.org/10.48546/workflowhub.workflow.548.2> | | |
|  | | |
| **ABC MD Setup** | Provides a pipeline to setup DNA structures for the Ascona B-DNA Consortium (ABC) members, following the work started with the NAFlex tool to offer a single, reproducible pipeline for structure preparation, ensuring reproducibility and coherence between all the members of the consortium | Ambertools[4] |
| **GitHub:** <https://github.com/bioexcel/biobb_wf_amber_md_setup> (abcsetup notebook)  **WorkflowHub**: <https://workflowhub.eu/workflows/196>  **bio.tools**: <https://bio.tools/bioexcel_building_blocks_tutorials_abc_md_setup>  **DOI**: <https://doi.org/10.48546/workflowhub.workflow.196.5> | | |
|  | | |
| **Structural Dna Helical Parameters** | Illustrates the process of extracting structural and dynamical properties from helical parameters of DNA MD trajectories | Curves+, Canal[17] |
| **GitHub:** <https://github.com/bioexcel/biobb_wf_dna_helparms>  **WorkflowHub**: <https://workflowhub.eu/workflows/195>  **bio.tools**: <https://bio.tools/bioexcel_building_blocks_tutorials_structural_dna_helical_parameters_from_md_trajectory>  **DOI**: <https://doi.org/10.48546/workflowhub.workflow.195.5> | | |
|  | | |
| **Molecular Structure Checking** | Illustrates the process of checking a molecular structure before using it as an input for a Molecular Dynamics simulation | Structure_Checking,  Ambertools[4] |
| **GitHub:** <https://github.com/bioexcel/biobb_wf_structure_checking>  **WorkflowHub**: <https://workflowhub.eu/workflows/775>  **bio.tools**: <https://bio.tools/bioexcel_building_blocks_tutorials_molecular_structure_checking>  **DOI:** <https://doi.org/10.48546/workflowhub.workflow.775.1> | | |
|  | | |


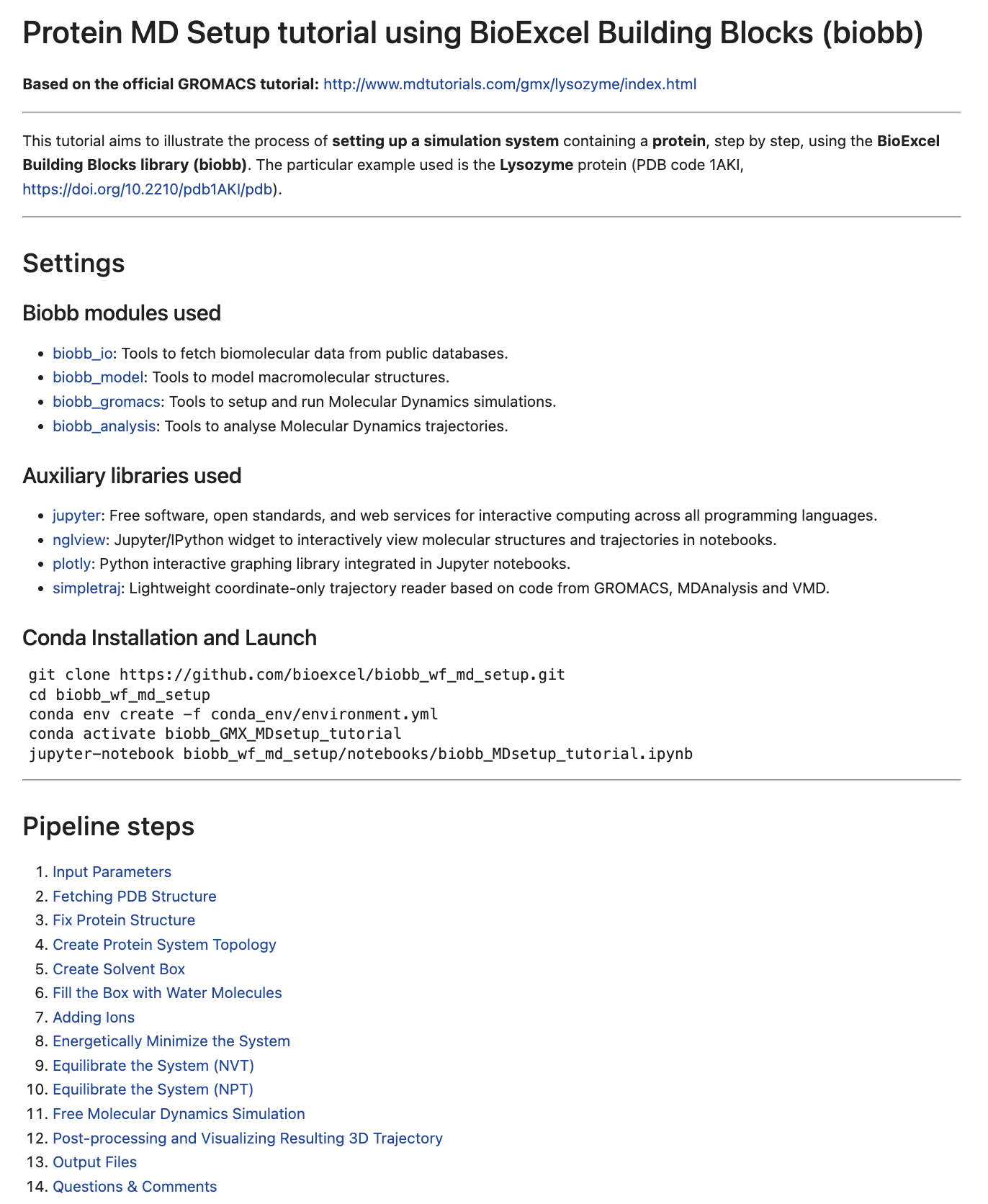


**Fig A**. Uniform header for all the BioBB tutorials, exemplified with the GROMACS Protein MD setup workflow. Sections included are i) title and description; ii) BioBB modules used; iii) auxiliary libraries used; iv) command lines required to install and launch the workflow; and v) pipeline steps.

**References**

1. Abraham, M.J., et al., *GROMACS: High performance molecular simulations through multi-level parallelism from laptops to supercomputers.* SoftwareX, 2015. **1–2**: p. 19-25.

2. Sousa da Silva, A.W. and W.F. Vranken, *ACPYPE - AnteChamber PYthon Parser interfacE.* BMC Research Notes, 2012. **5**: p. 367-367.

3. O'Boyle, N.M., et al., *Open Babel: An open chemical toolbox.* Journal of Cheminformatics, 2011. **3**(1): p. 33.

4. Case, D.A., et al., *The Amber biomolecular simulation programs.* J Comput Chem, 2005. **26**(16): p. 1668-88.

5. Gapsys, V., et al., *pmx: Automated protein structure and topology generation for alchemical perturbations.* Journal of Computational Chemistry, 2015. **36**(5): p. 348-354.

6. Trott, O. and A.J. Olson, *AutoDock Vina: improving the speed and accuracy of docking with a new scoring function, efficient optimization, and multithreading.* J Comput Chem, 2010. **31**(2): p. 455-61.

7. Armstrong, D.R., et al., *PDBe: improved findability of macromolecular structure data in the PDB.* Nucleic Acids Res, 2020. **48**(D1): p. D335-D343.

8. Le Guilloux, V., P. Schmidtke, and P. Tuffery, *Fpocket: An open source platform for ligand pocket detection.* BMC Bioinformatics, 2009. **10**(1): p. 168.

9. J, C., et al., *FlexServ: an integrated tool for the analysis of protein flexibility.* 2009.

10. de Groot, B.L., et al., *Prediction of protein conformational freedom from distance constraints.* Proteins: Structure, Function, and Bioinformatics, 1997. **29**(2): p. 240-251.

11. Laine, E. and S. Grudinin, *HOPMA: Boosting Protein Functional Dynamics with Colored Contact Maps.* The Journal of Physical Chemistry B, 2021. **125**(10): p. 2577-2588.

12. Lopéz-Blanco, J.R., J.I. Garzón, and P. Chacón, *iMod: multipurpose normal mode analysis in internal coordinates.* Bioinformatics, 2011. **27**(20): p. 2843-50.

13. Zhang, S., et al., *ProDy 2.0: increased scale and scope after 10 years of protein dynamics modelling with Python.* Bioinformatics, 2021. **37**(20): p. 3657-3659.

14. Meyer, T., et al., *Essential Dynamics:  A Tool for Efficient Trajectory Compression and Management.* J Chem Theory Comput, 2006. **2**(2): p. 251-8.

15. Gelpí, J.L., et al., *Classical molecular interaction potentials: improved setup procedure in molecular dynamics simulations of proteins.* Proteins, 2001. **45**(4): p. 428-37.

16. P, S., et al., *Exploration of conformational transition pathways from coarse-grained simulations.* 2013.

17. Blanchet, C., et al., *CURVES+ web server for analyzing and visualizing the helical, backbone and groove parameters of nucleic acid structures.* Nucleic Acids Res, 2011. **39**(Web Server issue): p. W68-73.
